# eCry1Gb.1Ig, A Novel Chimeric Cry Protein with High Efficacy against Multiple Fall Armyworm (*Spodoptera frugiperda*) Strains Resistant to Different GM Traits

**DOI:** 10.3390/toxins14120852

**Published:** 2022-12-03

**Authors:** Hyunsook Chae, Zhimou Wen, Travis Hootman, Jo Himes, Qianqian Duan, Joel McMath, Jesse Ditillo, Richard Sessler, Jared Conville, Ying Niu, Phillip Matthews, Fabricio Francischini, Fangneng Huang, Matthew Bramlett

**Affiliations:** 1 Syngenta Crop Protection LLC, Durham, NC 27709, USA; 2 Syngenta Biotechnology (China) Co., Ltd., Zhongguancun Life Science Park, Beijing 102206, China; 3 Department of Entomology, Louisiana State University Agricultural Center, Baton Rouge, LA 70803, USA; 4 Centro de Tecnologia Canavieira (CTC), Piracicaba 13400-970, SP, Brazil

**Keywords:** eCry1Gb.1Ig, engineered Cry proteins, fall armyworm control, corn and soybean pest control

## Abstract

*Spodoptera frugiperda* (fall armyworm, FAW) is one of the most devastating insect pests to corn and soybean production in the Americas and is rapidly expanding its range worldwide. It is known to be hard to control either by chemical insecticide applications or by GM. Although the use of GM traits can be an effective way to control this pest, it is very rare to find native insecticidal proteins that provide the necessary level of FAW control in crop fields where FAW pressure and damage are high. Insecticidal Cry proteins sourced from *Bacillus thuringiensis* have been heavily utilized in the development of crops with GM traits; however, it is increasingly difficult to identify Cry proteins with unique modes of action. Protein engineering via a phylogenetically guided Cry protein domain swapping approach enabled us to discover novel chimeric Cry proteins engineered from inactive parent sequences. Some of these chimeras show excellent efficacy against key biotypes of FAW from Brazil and North America. In this study, we characterized a Cry-based chimera eCry1Gb.1Ig that is a very potent FAW toxin. eCry1Gb.1Ig showed high efficacy against multiple FAW strains that are resistant to various traits, including Cry1Fa, Vip3Aa and Cry1A.105/Cry2Ab. These results clearly indicate that the FAW strains resistant to Cry1Fa, Vip3Aa or Cry1A.105/Cry2Ab demonstrate no cross-resistance to eCry1Gb.1Ig and strongly suggest that eCry1Gb.1Ig acts through a novel mode of action compared to the existing traits. In addition to its FAW activity, eCry1Gb.1Ig has also been shown to control *Chrysodeixis includens* (soybean looper, SBL) and *Anticarsia gemmatalis* (velvetbean caterpillar, VBC), which are significant pests of soybean. When eCry1Gb.1Ig was introduced into corn and soybean crops, transgenic events showed strong efficacy against FAW, SBL and VBC, but no adverse plant phenotypes. This suggests that the in planta expression of the eCry1Gb.1Ig protein does not compromise plant growth or reproduction and can protect plants from FAW-related damage. Therefore, this valuable discovery will provide a differentiating FAW control trait that will give growers another tool to help them reduce yield loss due to FAW.

## 1. Introduction

Global crop production faces many challenges in the effort to deliver successful annual harvests. These include abiotic stresses, such as drought and extreme temperatures, and biotic stresses, such as disease and insect pressure. Pest pressures are responsible for annual global losses up to 22% of annual corn production alone [1]. Unfortunately, these challenges, including pest pressure, are only being exacerbated by global climate change, which may shift overwintering populations of seasonally migratory insects to higher latitudes, and hence allow them to increasingly impact larger areas of agricultural production [2]. When combined with the long-appreciated propensity of key pest species to develop resistance against commercialized insecticides, this creates a dire need for powerful and new insect control products [3,4]. Genetically modified (GM) crops, such as soybean and corn varieties that incorporate insecticidal traits, are a critical tool for the control of many pest species, particularly those in the Lepidoptera order. These products have value in multiple global growing regions and their successful long-term use must be protected by the implementation of insect resistance management (IRM) strategies and product refreshment via the discovery and development of new insecticidal traits [5,6].

*Spodoptera frugiperda* (fall armyworm, FAW) has recently established itself as a Lepidopteran pest of global significance. In North America, FAW seasonally migrates northward from the continuously growing populations present in Texas and Florida and routinely reaches southern Canada by the growing season’s end. Native to the Americas and traditionally a pest only in this region, FAW has spread from the eastern United States and the Caribbean to Africa, through southern Asia, and within the last 3 years, has reached eastern and southeastern Asia [7,8,9]. This impressive expansion occurred over the span of 6 years and was aided by FAW being a strong flyer, possessing an expansive host plant range, and a lack of diapause [10,11].

Cry proteins sourced from *Bacillus thuringiensis* have been heavily utilized to control agricultural pests. Cry proteins are naturally occurring insecticidal proteins that are produced by the microbe as insoluble crystals of protoxins, which are solubilized once ingested by the insect. Upon solubilization, the protoxin is cleaved by gut proteases to produce a mature form. The mode of action is not fully understood, but the most likely mechanism is that the mature protein oligomerizes and binds to receptor proteins within sensitive insects to produce pores in gut cell membranes [12].

Regarding insecticidal GM corn products, to date, FAW has been controlled by the following five commercialized transgenic *Bacillus thuringiensis* (Bt) corn events: Bt11 and MON810, both of which express Cry1Ab, TC1507 that expresses Cry1Fa, MON89034 that expresses both Cry1A.105 and Cry2Ab2, and MIR162 that expresses Vip3Aa20. Bt11 and MON810 were approved in 1996, and while Cry1Ab still offers strong control of Lepidopteran borers, such as *Ostrinia nubilalis* (European corn borer, ECB), it is of limited current value in the control of FAW [13]. TC1507 was approved in 2001 and still controls limited populations of FAW, such as those in North America, although resistance in the United States has been identified [14]. It has, however, faced severe resistance pressure in other regions with FAW populations in Puerto Rico, developing high levels of resistance within 3 years of commercial growth [15]. This resistance allele confers markedly reduced susceptibility to Cry1Fa [16,17]. Similarly, the South American populations of FAW have developed resistance to Cry1Fa, which has spread rapidly to most of the corn- and soy-growing regions of Brazil. One prominent Cry1Fa resistance allele has been well characterized and involves a mutation to an ABC transporter protein (ABCC2), Cry1Fa’s functional receptor [18]. MON89034 was approved in 2007 and still offers control of FAW in many regions, but is similarly compromised in Brazil. Cry1A.105 in particular has been shown to suffer from cross-resistance to Cry1Fa and Cry1Ab [19]. Possessing two FAW active toxins, the example of MON89034 shows that even pyramided products can be overcome by this pest. MIR162 was approved most recently in 2008 and still provides effective control of FAW across different global growing regions, making it one of agriculture’s most valuable GM assets [20]. However, it is also under serious pressure and while low in frequency, resistance alleles are known to be present in multiple regions [21,22]. The risk to MIR162 is compounded by the fact that the Vip3Aa protein is utilized in multiple GM crops and due to the aforementioned resistance situation that is affecting all other maize GM events, MIR162 often acts as a single mode of action (MOA) against FAW. This situation translates to a critical need for new FAW MOAs and new pyramided GM products for both corn and soy.

According to the most likely mechanistic hypothesis, three-domain Cry proteins, such as Cry1Ab or Cry1Fa, function via binding to receptor proteins expressed on the surface of insect midgut cells [23]. Decades of research has revealed many, but not all, of the relevant MOA details. These toxins are natively expressed as a protoxin that consists of seven domains, which are cleaved within the insect’s gut to produce an active core of three domains [24]. Domains 2 and 3 are both known to be involved in receptor binding and are the key mediators of differentiating binding sites (site of action).

In 1996, it was first reported that chimeric Cry proteins produced via third domain swaps could drastically change the insecticidal properties of a given toxin [25]. De Maagd showed that a Cry1Ab template protein with a swapped Cry1Ca third domain created a new protein (HO4) that exhibited increased activity versus *Spodoptera exigua*, reaching levels greater than those observed with either parent protein. The commercial relevance of this approach was observed again later with Cry1A.105, present in MON89034, which combines a Cry1Ab template with a swapped Cry1Fa third domain. Notably, this approach has been extended beyond targeting Lepidopteran pests with the release of the eCry3.1Ab toxin, present in the *Diabrotica virgifera virgifera* active corn event SYN05307. This example is remarkable, as it combined an active parent protein, mCry3A, as the template with a swapped inactive protein third domain from Cry1Ab [26].

In this report, we describe the novel engineered protein eCry1Gb.1Ig, which was generated by the further development of the chimera approach using phylogenetically distinct and weakly active parent donors. eCry1Gb.1Ig is highly efficacious against multiple FAW strains with resistance to Cry1Fa, Vip3Aa and Cry1Ab.105/Cry2Ab, respectively. This suggests that eCry1Gb.1Ig has a distinctive MOA and will be an important tool in the control of global FAW and other Lepidopteran pests.

## 2. Results

### 2.1. Generation of Cry1Gb-Based Chimeric Proteins

**Figure 1:**
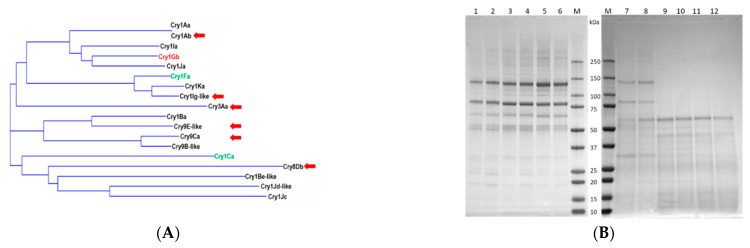
Cry protein engineering through domain 3 swapping. (**A**) Phylogenetic analysis of domain 3 sequences of native *Cry* genes. Domain 3 sequences indicated by arrows were selected for domain swapping. (**B**) SDS-PAGE analysis of Bt crystal protein production of engineered chimeric genes. Crystal proteins from two independent Bt clones of each chimeric gene were analyzed via SDS-PAGE. M: protein molecular weight markers; lane 1 and 2: Cry1Gb-3A chimera; lane 3 and 4: eCry1Gb.1Ig chimera; lane 5 and 6: Cry1Gb-9E chimera; lane 7 and 8: Cry1Gb-1Ab chimera; lane 9 and 10: Cry1Gb-8D chimera; and lane 11 and 12: Cry1Gb-9Ca chimera.

In order to engineer three-domain Cry proteins through domain swapping, the amino acid sequence similarity of domain 3 across numerous native Cry proteins was analyzed and a phylogenetic tree was generated with a set of the most diverse sequences (Figure 1A). For the sequence analysis, we used publicly available native Cry sequences, as well as newly discovered native genes from our internal discovery pipeline. We hypothesized that domain 3 swapping across a wide range of Cry protein subfamilies is a feature of Cry protein evolution that can extend or generate target specificity. To create new FAW activity based on this hypothesis, we attempted to accelerate domain 3 mediated evolution of Cry proteins by utilizing the most diverse domain 3 sequences selected from various clades of the phylogenetic tree (Figure 1A), with the intention to increase sequence diversity of the resulting chimeric proteins.

**Figure 2:**
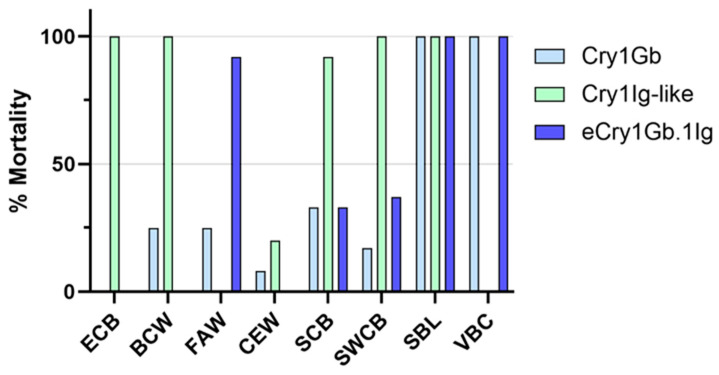
Insecticidal spectrum screening of the chimeric protein eCry1Gb.1Ig and its parental proteins, Cry1Gb and Cry1Ig-like. Mortalities of various Lepidopteran insects were assessed using L1 larvae infested with eCry1Gb.1Ig, Cry1Gb, or Cry1Ig-like via a diet-based insect bioassay. CEW = corn earworm, ECB = European corn borer, BCW = black cutworm, FAW = fall armyworm, SCB = sugarcane borer, SWCB = southwestern corn borer, SBL = soybean looper, and VBC = velvetbean caterpillar.

In general, Cry proteins with strong insecticidal activity against FAW are rare in native Bt genomes. We found that only a few native Cry proteins, including Cry1Gb from our discovery activities, showed low but measurable FAW efficacy at the insecticidal activity screening stage (Figure 2). When the domain 3 sequence of Cry1Gb was compared to other Cry sequences, we found that it is similar to domain 3 of Cry1Ja, which belongs to a different Cry protein subfamily compared to Cry1Gb as whole proteins. Using Cry1Gb as a template, six recombinant genes were generated by swapping domain 3 of Cry1Gb with a set of domain 3 sequences selected from the previous phylogenetic sequence analysis (Table 1). Then, chimeric proteins were produced using a Bt crystal protein expression system. Four out of the six recombinant genes (*cry1Gb-1Ab*, *eCry1Gb.1Ig*, *cry1Gb-9E* and *cry1Gb-3A*) produced chimeric proteins of expected size, whereas the remaining two recombinant genes (*cry1Gb-8D* and *cry1Gb-9Ca*) did not (Figure 1B).

### 2.2. Screening of Cry1Gb Chimeric Proteins for Insecticidal Activities against FAW and Other Insects

Several screening bioassays were conducted to evaluate the four chimeric proteins of expected size for their insecticidal activities against FAW and other insects. Our objective for the first insecticidal activity screen was to evaluate if any of these chimeric proteins possessed FAW activity. We targeted the Bt-susceptible FAW strain BenFAW by exposing neonates to chimeric proteins generated from two independent Bt clones (a and b) for 7 days and recording larval mortalities. At an arbitrarily chosen diagnostic concentration of 3.2 μg/cm^2^, the Cry1Gb-1Ab chimera demonstrated no FAW activity for either of the treatments, but Cry1Gb-9E and Cry1Gb-3A chimeras showed mild insecticidal activities with mortalities ranging from 8% to 33%, depending on the Bt clones used for chimeric protein production (Table 2). In contrast, the eCry1Gb.1Ig chimera showed strong insecticidal activity; 100% or 83% of BenFAW larvae were killed by eCry1Gb.1Ig at the same concentration of 3.2 μg/cm^2^ prepared from two independent Bt clones (Table 2). In addition, the remaining larvae were severely retarded in growth or moribund, suggesting that eCry1Gb.1Ig is very efficacious against FAW. These results indicate that a new FAW active chimeric protein was created by domain 3 swapping, using Cry1Gb as a template.

Our objective for the next round of screening bioassays was to evaluate the potential insecticidal spectrum of eCry1Gb.1Ig. We tested eCry1Gb.1Ig and its parental proteins, Cry1Gb and Cry1Ig-like, with neonates of various Lepidoptera species. As shown in Figure 2, the chimeric protein demonstrated new FAW activity when compared to its two parental proteins, which displayed negligible or no FAW activity at a concentration of 2 µg/cm^2^. eCry1Gb.1Ig also maintained the activity of its parental protein, Cry1Gb, against *Diatraea saccharalis* (sugarcane borer, SCB), *Diatraea grandiosella* (southwestern corn borer, SWCB), *Chrysodeixis includens* (soybean looper, SBL) and *Anticarsia gemmatalis* (velvetbean caterpillar, VBC). On the other hand, the observed Cry1Ig-like activity against ECB, *Agrotis ipsilon* (black cutworm, BCW), SCB, and SWCB was not passed on to the chimera. When eCry1Gb.1Ig was tested at a concentration of 2 µg/cm^2^ against other *Spodoptera* species, it also showed strong efficacy with 100% mortality for *Spodoptera litura* (Chinese biotype) and 46% mortality for *Spodoptera cosmioides* (Brazilian biotype) (Table 3).

The above observations support the idea that the activity spectrum of chimeric proteins heavily relies on that of their template proteins. Therefore, the discovery of new FAW activity in the eCry1Gb.1Ig chimera suggests that domain 3 swapping is a feature of the natural evolutionary process that may be used to extend or stimulate new insecticidal activity of its parental proteins.

### 2.3. eCry1Gb.1Ig Is Highly Efficacious against FAW Strains Resistant to Cry1Fa, Vip3Aa and Cry1A.105/Cry2Ab2

To evaluate the possibility of cross-resistance between eCry1Gb.1Ig and Cry1Fa or Vip3Aa, we implemented susceptibility screening with eCry1Gb.1Ig against Cry1Fa-resistant and Vip3Aa-resistant FAW strains. The eCry1Gb.1Ig chimera showed strong insecticidal activity against both susceptible and resistant FAW strains at a concentration of 2 µg/cm^2^, suggesting no cross-resistance between the proteins (Table 4). We then determined the potency of eCry1Gb.1Ig against two susceptible and multiple resistant FAW strains by generating their EC_50_ values (Table 5). eCry1Gb.1Ig is highly efficacious against not only the two susceptible strains, but also the three strains resistant to Cry1Fa, Vip3Aa and Cry1A.105/Cry2Ab2, respectively. eCry1Gb.1Ig had an EC_50_ value of 2.92 ng/cm^2^ for Cry1Fa-resistant BrFAW and 3.57 ng/cm^2^ for susceptible BenFAW when neonates of the two strains were exposed to the protein for 5 days. The 95% confidence interval overlapped for the two EC_50_ values, suggesting that eCry1Gb.1Ig was similarly toxic to the two strains. When neonates of Cry1A.105/Cry2Ab2-resistant FAW_DUAL_R and susceptible FAW_LSU_S were exposed to eCry1Gb.1Ig for 7 days, the protein also showed similar toxicity to the two strains. The EC_50_ value of eCry1Gb.1Ig was 16.9 ng/cm^2^ for FAW_DUAL_R and 12.6 ng/cm^2^ for FAW_LSU_S with overlapping 95% confidence intervals. The 5-day EC_50_ value of eCry1Gb.1Ig for Vip3Aa-resistant FAW_Vip3A_R (54.6 ng/cm^2^) generated from a non-isogenic biotype of BenFAW was slightly higher than that of BenFAW, but eCry1Gb.1Ig was still very potent against the strain. Our results clearly indicated that eCry1Gb.1Ig demonstrates no cross-resistance to Cry1Fa, Vip3Aa or Cry1A.105/Cry2Ab2 and strongly suggest that eCry1Gb.1Ig is a novel insecticidal protein that controls FAW through an MOA that is different from the MOAs of Cry1F, Vip3A and Cry1A.105/Cry2Ab2.

### 2.4. Plants Carrying the eCry1Gb.1Ig Trait Effectively Control Not Just Susceptible, but Also Multiple Resistant FAW Strains

**Figure 3:**
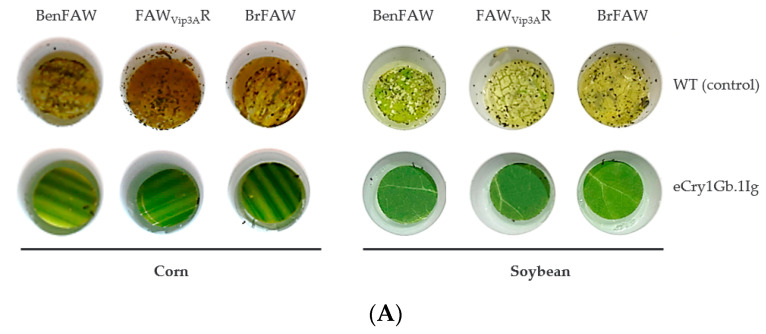

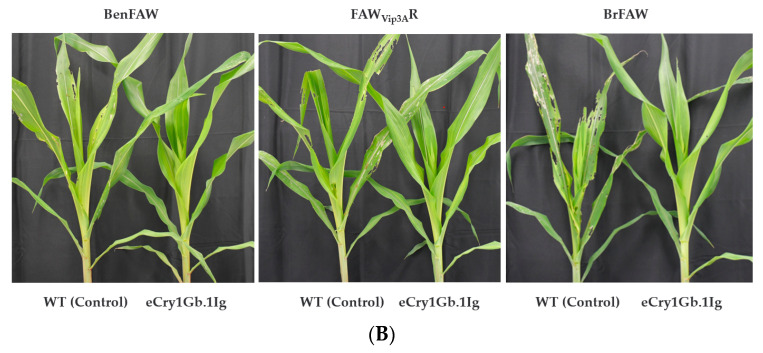
FAW feeding assays with eCry1Gb.1Ig-expressing corn events (**A**,**B**) and eCry1Gb.1Ig-expressing soybean events (**A**). A: leaf disc feeding assays. One representative feeding assay result with corn and soybean events carrying *eCry1Gb.1Ig.* Leaf discs of transgenic plants were infested with insects for 3 days. (**B**). On-plant feeding assays. One representative on-plant feeding assay result of a transgenic corn event carrying *eCry1Gb.1Ig.* Transgenic plants were infested with insects for 14 days. BenFAW: susceptible FAW strain; FAW_Vip3A_R: Vip3Aa-resistant strain; and BrFAW: Cry1Fa-resistant strain.

To assess the potential of eCry1Gb.1Ig as an insect control trait in plants, both corn and soybean transgenic events were initiated to express the eCry1Gb.1Ig protein driven by constitutive plant promoters. Figure 3A shows the representative results of leaf disc bioassays using a corn transgenic event against three different FAW strains (susceptible BenFAW, Vip3A-resistant FAW_Vip3A_R and Cry1Fa-resistant BrFAW). In comparison with the non-transgenic control plants, where each leaf disc was almost completely consumed by three larvae of any of the three FAW strains when infested for three days, the eCry1Gb.1Ig-expressing corn event was well protected, with almost no damage observed when similarly challenged (Figure 3A) and the observation was consistent across multiple replicates of three independent corn events (Supplementary Figure S1). We observed that larvae were either killed or significantly stunted when feeding on leaf discs from eCry1Gb.1Ig-expressing corn events. The protection of corn plants that express eCry1Gb.1Ig was further confirmed by on-plant feeding assays when each V4 stage plant was challenged with 20 FAW larvae for 14 days. As shown in Figure 3B, plants of a representative corn event harboring eCry1Gb.1Ig were healthy and had no visible damage after infestation of either susceptible or resistant FAW strains. A soybean transgenic event that carried the eCry1Gb.1Ig trait was also tested via leaf disc bioassay and the eCry1Gb.1Ig-expressing soybean event offered similar protection from feeding damage by the three FAW strains (Figure 3A). We also observed strong protection from the FAW strains with additional independent soybean events (Supplementary Figure S2). Our results demonstrate that eCry1Gb.1Ig is an efficacious FAW insecticidal protein in plants. Corn and soy plants that carry the eCry1Gb.1Ig trait can effectively control not just susceptible, but also multiple resistant FAW strains via a novel MOA.

## 3. Discussion

Although several Cry proteins, including Cry1Ca, Cry1Da, Cry1Fa and Cry9Ca, have been reported as active insecticidal proteins against multiple *Spodoptera* species, only a few, such as Cry1Fa and Cry1Ca, with efficacy against FAW hold current significance for agricultural application [27]. The Cry1G subfamily, with only three gene groups (*cry1Ga*, *cry1Gb* and *cry1Gc*) identified so far, is much smaller than the Cry1A subfamily and none of the Cry1G proteins are known to have significant insecticidal activity against the *Spodoptera* species [28,29]. A native Cry1Gb identified through our discovery activity has 97.4% amino acid sequence identity to Cry1Gb2 and showed negligible activity against FAW in our initial insect bioassays. By comparing domain sequences of various Cry proteins, we found that the domain 3 sequence of Cry1Gb shows high sequence identity with that of Cry1Ja and Cry1Ia, which demonstrate low or no activity against FAW, whereas the protein shares only a low level of identity with Cry1Fa and Cry1Ca in domain 3 sequences. In addition, the domain 2 sequence of Cry1Gb demonstrates very low similarity to that of Cry1Fa and Cry1Ca (32% and 37%, respectively), suggesting that Cry1Gb has a MOA that is different to that of Cry1Fa and Cry1Ca with regard to insecticidal activity.

The native Cry1Ig-like protein, which possesses high sequence similarity (91.3%) to Cry1Ig1, did not demonstrate any FAW activity based on our artificial insect diet assay. Its domain 3 shares only 57% identity with that of Cry1Gb and 38% with that of Cry1Ca. Although domain 3 of the Cry1Ig-like protein belongs to a clade with Cry1Fa and Cry1Ka in the phylogenetic tree, its sequence is still distinct and demonstrates 87% sequence similarity to the two sequences.

Based on the insecticidal spectrum of the Cry1Gb chimeric proteins generated, it is clear that insecticidal activity for specific chimeric proteins cannot be easily predicted because both additive and deleterious alterations in toxin activity were observed. This reinforces the view that both domains II and III contribute to insect midgut binding specificity; hence, new combinations of these two domains can generate new sites of action. This can be observed in the dramatic example of the eCry1Gb.1Ig chimera versus FAW, in which significant activity was demonstrated in the chimera that did not exist in either parent, i.e., Cry1Gb and Cry1Ig-like. The new and unique insecticidal effect of eCry1Gb.1Ig on FAW became more apparent when its wider insecticidal spectrum was examined. In every other tested insect species, the existing activity was either negatively affected or unchanged by the production of the chimera. The strong borer activity observed for the Cry1Ig-like protein was severely compromised in eCry1Gb.1Ig. In addition, the existing activity versus BCW and *Helicoverpa zea* (corn earworm, CEW) in both parental proteins was similarly absent in their progeny. We hypothesize that it is much easier to introduce detrimental effects than positive ones. This may explain why eCry1Gb.1Ig has a slightly narrower spectrum than its parental proteins. Nevertheless, eCry1Gb.1Ig maintains its activity towards key crop pests, such as SBL and VBC. In addition, it demonstrates significant activity against other *Spodoptera* species of agricultural concern, including *S. cosmoides* (Brazilian biotype) and *S. litura* (Chinese biotype). This spectrum of activity ensures that eCry1Gb.1Ig will be valuable as a GM insect control trait in multiple regions globally. 

The generation of new traits with different MOAs is especially important due to the development of insect populations that are resistant to commercial products. The ability of a new trait to control a resistant insect population is an unambiguous indicator that the tested toxin utilizes a different MOA, which is likely (although not always) to be mediated through differences in the midgut binding site (site of action). By using diet-based assays and/or plant-based feeding assays, we have clearly demonstrated in this study that eCry1Gb.1Ig is highly efficacious against FAW strains with resistance to the Bt corn traits currently on the market. Two strains possess resistance to the Cry1Fa-containing event TC1507, with one population originating from Brazil and the other from Puerto Rico. The primary Cry1Fa resistance mechanisms at work in these two geographical regions have been well characterized and are likely, although not confirmed, to be at work in the populations specifically tested in this study [17,18]. Another strain possesses resistance to the Cry1A.105/Cry2Ab-containing dual MOA event MON89034 and the fourth possesses lab-selected resistance to Vip3Aa [30,31]. eCry1Gb.1Ig is highly efficacious against all four resistant strains, clearly demonstrating that FAW populations resistant to the proteins that are expressed in all currently marketed FAW active insect control GM corn events have no cross-resistance to eCry1Gb.1Ig. These results strongly suggest that eCry1Gb.1Ig kills FAW by a unique MOA.

It is hard to overstate the impact and importance of new FAW active GM traits. Since the beginning of the GM age, only four events have been broadly commercialized. All but one of these are facing severe challenges in controlling FAW, due to the emergence of resistant field populations. New traits such as eCry1Gb.1Ig will form part of the much-needed next generation of FAW active products. When pyramided with other insect control traits, Cry1Gb.1Ig will help to ensure that growers have the tools to adequately protect their harvest from devastating pests, such as FAW.

## 4. Materials and Methods

### 4.1. Recombinant DNA and Bt Protein Production

Protein sequences of the following native Cry proteins were collected from public databases, including GenBank, as well as Syngenta internal discovery: Cry1Aa (GenBank accession number AAA22353), Cry1Ab (GenBank accession number AAA22330), Cry1Ia (GenBank accession number CAA44633), Cry1Gb (GenBank accession number OP874794), Cry1Ja (GenBank accession number AFP33257), Cry1Fa (GenBank accession number AAA22348), Cry1Ka (GenBank accession number AAB00376), Cry1Ig-like (GenBank accession number OP874795), Cry3Aa (GenBank accession number AAA22336), Cry1Ba (GenBank accession number CAA29898.1), Cry9E-like (GenBank accession number OP874791), Cry9Ca (GenBank accession number OP874790), Cry9B-like (GenBank accession number OP874792), Cry1Ca (GenBank accession number AF362020.1), Cry8Db (GenBank accession number BAF93483.1), Cry1Be-like (GenBank accession number OP874793), and Cry1Jc (GenBank accession number OP874789). Vector NTI software was used to derive the phylogenetic tree of Cry domain 3 sequences. The DNA sequences that encoded the six chimeric proteins were generated in silico by swapping domain 3 sequences of Cry1Gb with those of other Cry proteins, including eCry1Gb.1Ig (GenBank accession number OP837419), Cry1Gb-3A (GenBank accession number OP837420), Cry1Gb-9E (GenBank accession number OP837421), Cry1Gb-1Ab (GenBank accession number OP837422), Cry1Gb-8D (GenBank accession number OP837423) and Cry1Gb-9Ca (GenBank accession number OP837424). cDNAs of native and engineered chimeric Cry proteins were synthesized and their sequences were verified by Genewiz (South Plainfield, NJ, USA), then cloned into a Bt expression vector that contained a Cry1Ac promoter and no terminator. The plasmid DNAs were introduced into Bt strain AB227 via electroporation-mediated transformation, following a procedure described by [32]. Except for the Vip3Aa19 protein, which was produced in *E. coli* (BL21DE3) and purified using Q Sepharose FF columns and then dissolved in PBS, all other native and engineered proteins were produced in Bt as crystal proteins, which were then purified from the cultures and dissolved in Buffer 1 that contained 50 mM Na_2_CO_3_ (pH 11.0) and 2 mM DTT. Purified proteins were quantified using BCA assay and some were also evaluated on SDS-PAGE, before they were used in artificial bioassays [33].

### 4.2. Insects

Six FAW strains were used in bioassays to evaluate the chimeric proteins generated from protein engineering for their insecticidal activities against FAW. BenFAW, purchased from Benzon Research (Carlisle, PA, USA), originated from the USDA laboratory in Stoneville (Mississippi). Benzon received the strain from a now non-existent third party about 20 years ago and has maintained the colony in its facility without infusion of new genetic materials or exposure to any insecticides. The BrFAW colony was established from larvae collected in MON89034-containing corn fields around Lucas do Rio Verde (Mato Grosso, Brazil) during the 2016/2017 corn growing season. A subpopulation of the BrFAW colony has been maintained since 2017, without exposure to any insecticides. FAW_Vip3A_R is a FAW strain selected by exposing FAW neonates to Vip3Aa19 in the laboratory for multiple generations. Toxicity studies indicated that BrFAW was highly resistant to Cry1Fa and FAW_Vip3A_R was highly resistant to Vip3Aa, with the resistance levels being stable in both strains (Wen et al., unpublished). Cry1Fa-R-PR was established in 2009 after being collected from non-trait corn fields near Salinas, Puerto Rico and the strain was more than 200-fold resistant to Cry1Fa (not published). FAW_LSU_S, established from insects collected from non-Bt maize fields in Franklin Parish, Louisiana, USA, in 2016, has been documented to be susceptible to several Bt toxins, including Cry1Fa, Cry1Ab.105, Cry2Ab2 and Vip3Aa [30,34,35]. FAW_DUAL_R was established from the F_2_ generation of reciprocal crossing between a Cry1A.105-resistant strain and a Cry2Ab2-resistant strain of FAW. FAW_DUAL_R has been documented to be able to survive and complete larval development on transgenic Bt plant tissues that expressed both Cry1A.105 and Cry2Ab2 proteins [30,35]. Multiple other insects, including CEW, ECB, BCW, SCB, SWCB, SBL, and VBC, were also purchased from Benzon to test FAW-active proteins for a possible expanded spectrum. Similar to BenFAW, these insects have been reared without infusion of new genetic materials or exposure to any insecticides for more than 10 years. In addition, three other *Spodoptera* species, with two from Brazil (*S. eridania,* BR and *S. cosmioides,* BR) and one from China (*S. litura*, CN), were used in the spectrum tests. For all insect species, the neonates that hatched within 24 h were used in bioassays.

### 4.3. Diet Bioassays

Three types of diet bioassay methods were used in this study. The Type 1 method was a surface-coating bioassay using 24-well plates, with each well containing 1 mL of multiple species diet prepared according to the vendor’s instructions (Southland Products, Lake Village, AR, USA). The surface of the solidified diet in each well was evenly coated with a thin layer of a protein, as described below. First, 60 µL of protein at a desired concentration was pipetted into each well of a plate; second, the plate was placed on an New Brunswick^TM^ Innova® 2100 platform shaker (Eppendorf North America, Enfield, CT, USA) for shaking at a speed of 120 RMP. Fans were used to gently blow horizontally over the plates to accelerate the coating process and plates were monitored to make sure that the diet surface was dried but free of cracks. The same volume of buffer was used to coat each of the control wells. The Type 2 method also used 24-well plates and the diet was prepared as in the Type 1 method, i.e., each well contained 1 mL of diet. The diet in each well was first dried using lyophilization and proteins at a desired concentration and a buffer was then added to soak the dried diet plug. The protein- or buffer-soaked diet was again lyophilized for later use. Immediately before insect infestation, 1 mL of pure water was added to each well to rehydrate the diet plug that contained a protein toxin or buffer. For both Type 1 and Type 2 bioassays, a piece of Breathe-Easy^®^ film (USA Scientific) was used to cover each plate after insect infestation. The Type 3 method used 32-cell plates (C-D International, Pitman, NJ, USA) for determination of eCry1Gb.1Ig toxicity against FAW_LSU_S and FAW_DUAL_R.

To screen the four chimeric proteins (Cry1Gb-3A, eCry1Gb.1Ig, Cry1Gb-9E, and Cry1Gb-1Ab) for insecticidal activities against the known susceptible BenFAW strain reported in Table 2, the Type 1 method was used to test each chimeric protein twice. Protein for each test was prepared from an independent clone. Depending on the amount, each protein at a fixed concentration of 3.2 µg/cm^2^ was tested using 12 or 24 BenFAW neonates with 1 larva per well. Cry1Fa at a concentration of 1 µg/cm^2^ served as a reference for screening the four chimeric proteins. The Type 1 method was also similarly used for screening the potential spectrum of eCry1Gb.1Ig and its parental proteins reported in Figure 2, for screening *S. litura*, CN, as reported in Table 3, and screening the insecticidal activity of eCry1Gb.1Ig against susceptible and resistant FAW strains, as reported in Table 4. To screen the insecticidal activities of eCry1Gb.1Ig against *S. eridania*, BR, and *S. cosmioides*, BR, the Type 2 method was used, where 500 µL of eCry1Gb.1Ig at a concentration of 60 µg/mL or equal volume of buffer was added to each diet plug. The same volume of Vip3Aa19 at a concentration of 60 µg/mL in PBS served as a reference for screening eCry1Gb.1Ig. For all the screening bioassays described above, plates were stacked and placed in the dark at room temperature. Seven days after neonate infestation, the assay plates were examined to record mortality. For screening the four chimeric proteins, insect growth was also observed.

To determine the toxicity of eCry1Gb.1Ig to BenFAW, BrFAW and FAW_Vip3A_R strains, the Type 1 bioassay method was used to calculate EC_50_ values by Probit analysis (version 1.5, Environmental Protection Agency (EPA), Washington, DC, USA). EC_50_ was the concentration of eCry1Gb.1Ig that caused a specific effect in 50% of the insects tested, where the specific effect was defined as either being dead or failure to advance to the 2nd instar in 5 days. At least three replicates of bioassays were executed for each combination of FAW strain and Bt concentration with the following eCry1Gb.1Ig concentrations prepared in 50 mM sodium carbonate (pH 11.0, Teknova): 0 (buffer only), 0.1, 0.3, 1, 3, 10, 30, 100, 300, 500, 1000 and 3000 ng/cm^2^. For each replicate, 24 neonates were exposed and all bioassay plates were placed in an incubator set at 27 °C and 14:10 h (L:D). Since we observed that it took 1.5 to 3 days for the BrFAW neonates fed with the plain diet to advance to the second instar under the above conditions, the specific effect was recorded 5 days after exposure of the neonates to a protein. The concentration series of eCry1Gb.1Ig was chosen based on the preliminary bioassay results, where (1) the specific effect similar to that caused by the buffer treatment was observed for at least one eCry1Gb.1Ig concentration in the low concentration range; (2) the specific effect in 100% or close to 100% of insects was observed for at least one concentration in the high concentration range; and (3) the in-between specific effect was observed for at least three concentrations in the middle range. For confirmation of resistance in BrFAW to Cry1Fa and FAW_Vip3A_R to Vip3Aa19 when the two strains were assayed for their susceptibility to eCry1Gb.1Ig, at least three replicates of bioassays were also conducted for these two strains against the corresponding Cry1Fa or Vip3Aa19 strains at the concentrations of 0, 1000 and 3000 ng/cm^2^. 

To determine the toxicity of eCry1Gb.1Ig to FAW_LSU_S and FAW_DUAL_R strains, the Type 3 bioassay method was used. Appropriate Bt solutions used in the bioassays were prepared by adding the Bt protein (in a buffer) to distilled water that contained 0.1% Triton-X100. In each bioassay, there were seven Bt concentrations, ranging from 6.67 to 6670 ng/cm^2^, plus a negative control treated with +0.1% Triton X100 buffer only and a blank control treated with 0.1% Triton-X 100. In the assays, 0.8 mL of the above-mentioned Southland diet was placed into each cell of the 32-cell plates using 20 mL syringes (Becton, Dickinson and Company, Franklin Lakes, NJ, USA). After the diet in the cells solidified, an amount of 50 μL of a control or Bt solution was applied to the diet surface in each cell. The plates were then shaken by hand to ensure the Bt solution uniformly covered uniformly the diet surface. After the solution on the diet surface dried, one neonate (<24 h) of an insect strain was released on the diet surface in each cell. After larval inoculation, cells were covered with vented lids (C-D International, Pitman, NJ, USA). The bioassay plates were placed in an environmental chamber maintained at 26 °C, 50% RH, and for a 16:8 (L:D) h photoperiod. Larval mortality and growth inhibition were recorded on the 7th day after inoculation. Each combination of insect strains with different protein concentrations was replicated 4 times, with 32 larvae in each replicate. The 7-day EC_50_ values of eCry1Gb.1Ig against FAW_LSU_S and FAW_DUAL_R were similarly determined, where the specific effect was defined as either being dead or failure to advance to the 3^rd^ instar in 7 days.

### 4.4. Leaf Disc and on-Plant Feeding Bioassays

Leaf disc feeding assays and/or on-plant feeding assays were conducted using transgenic plants that expressed eCry1Gb.1Ig. Corn plants were individually grown in 4” pots to the V4 stage and soybean in 3” pots to the V3–V4 stage for feeding assays. Plants were sampled for confirmation of eCry1Gb.1Ig expression in leaves by ELISA, before being used for the feeding assay setup. For the leaf-disc feeding assays, leaf discs were harvested using a 1/2” J-Cut tissue puncher (MIDCO Global, Kirkwood, MO, USA) and 128-well bioassay trays from Frontier Agricultural Sciences (Frontier, BAW128, Newark, DE, USA) were used following the method of Little et al., as briefly described below [36]. After each assay well was prefilled with 1 mL of 1% molten agarose and allowed to cool to room temperature, a single leaf disc per well was placed in the wells, with the abaxial surface touching the agarose. Two leaf discs from each plant, four plants for each eCry1Gb.1Ig event and three events for either soybean or corn were used for the feeding assay experiments. Three neonates were used to infest each leaf disc in a well and the assay wells were covered with 16-cell lids from Frontier (BACV16). The bioassay trays were placed at room temperature with natural light and examined 3 days post-neonate infestation.

On-plant feeding assays were also conducted for corn plants, using each of 3 FAW strains with 20 neonates that were used to infest the whorl area of each of 4 plants. These four plants included one plant that was negative and one each from the three events that expressed eCry1Gb.1Ig. The four plants were placed in a nylon insect cage (60 × 60 × 180 cm) purchased from MegaView Sciences (BD6E630) in a square where the leaves of each plant would touch the leaves of at least two other plants. The plants were arranged in such a way that the larvae could move freely among the plants in the cage. The cages that contained FAW-infested plants were placed in a plant growth chamber under the following conditions: light intensity at 650 µmols, photoperiod of 14 h light and 10 h dark, CO_2_ at 400 ppm and temperature at 26.7 °C for day and 21.1 °C for night. The on-plant feeding assay experiments lasted for 14 days.

## 5. Patents

Syngenta Crop Protection AG has at least one patent family related to the work reported in this manuscript.

## Figures and Tables

**Table 1:** List of chimeric Cry proteins generated via domain swaps that incorporated diverse domain 3 sequences.

Chimeric Proteins	Domain 1	Domain 2	Domain 3	Protoxin-Tail
Cry1Gb-3A	Cry1Gb	Cry1Gb	Cry3A	Cry1Gb
eCry1Gb.1Ig	Cry1Gb	Cry1Gb	Cry1Ig	Cry1Gb
Cry1Gb-9E	Cry1Gb	Cry1Gb	Cry9E	Cry1Gb
Cry1Gb-1Ab	Cry1Gb	Cry1Gb	Cry1Ab	Cry1Gb
Cry1Gb-8D	Cry1Gb	Cry1Gb	Cry8D	Cry1Gb
Cry1Gb-9Ca	Cry1Gb	Cry1Gb	Cry9Ca	Cry1Gb

**Table 2:** Insecticidal activity screening of Cry1Gb-based chimeric proteins against BenFAW, a known Bt-susceptible FAW strain.

Proteins	Concentrations (µg/cm^2^)	Mortality (%), a *	Mortality (%), b *
Cry1Gb-3A	3.2	25	33
eCry1Gb.1Ig	3.2	100	83
Cry1Gb-9E	3.2	8	17
Cry1Gb-1Ab	3.2	0	0
Cry1Fa	1.0	100	-
Buffer 1	0	8	-

* a, b: chimeric proteins produced from independent Bt clones; “-“: not determined. Buffer 1: sodium carbonate buffer (pH 11.0) with 2 mM DTT.

**Table 3:** Insecticidal activity screening of eCry1Gb.1Ig chimeric protein against various *Spodoptera* species.

Proteins	Mortality (% Effective Mortality)		
	*S. eridania,* BR	*cosmioides,* BR	*S. litura,* CN
eCry1Gb.1Ig	8	46	100
Vip3Aa19	100	100	-
Buffer 1	13	4	17
PBS	4	4	-

Efficacy against various *Spodoptera* species was assessed based on mortality of L1 larvae infested with chimeric proteins via a diet-based insect bioassay. BR: Brazilian biotype; CN: Chinese biotype; Buffer 1: sodium carbonate buffer (pH 11.0) with 2 mM DTT; PBS: phosphate-buffered saline, pH 7.2; and ”-“: not determined.

**Table 4:** Insecticidal activity screening of eCry1Gb.1Ig chimeric protein against susceptible and resistant FAW strains.

Proteins	FAW Mortality (% Effective Mortality)			
	BenFAW	FAW_Vip3A_R	Cry1Fa-R-PR	BrFAW
eCry1Gb.1Ig	100	100	100	100
Cry1Fa	100	100	0	8
Vip3Aa19	100	0	100	100
Buffer 1	0	0	0	0
PBS	0	0	0	0

Efficacy against various Bt resistant FAW strains was assessed based on effective mortality of L1 larvae infested with chimeric proteins (2 μg/cm^2^) via diet-based insect bioassay. Forty-eight insects were used in the screening bioassay for each toxin or buffer per insect strain. Buffer 1: sodium carbonate buffer (pH 11.0) with 2 mM DTT; PBS: phosphate-buffered saline, pH 7.2. BenFAW: a susceptible FAW strain; FAW_Vip3A_R: a Vip3A-resistant FAW strain; Cry1Fa-R-PR: a Cry1Fa-resistant strain; and BrFAW: a Cry1Fa-resistant strain that was established from larvae collected in MON89034-containing corn fields in Brazil.

**Table 5:** Toxicity of eCry1Gb.1Ig against susceptible and resistant FAW strains.

FAW Strains	Cry1Fa		Vip3Aa19		eCry1Gb.1Ig	
	No. Insects *	EC_50_ (95%CI)	No. Insects *	EC_50_ (95%CI)	No. Insects *	EC_50_ (95%CI)
BenFAW	816	72.8 (46.4–107)	912	39.0 (31.5–47.5)	792	3.57 (2.66–4.7)
BrFAW	216	>>3000			1080	2.92 (2.3–3.64)
FAW_Vip3A_R			360	>>3000	1152	54.6 (41.5–70.2)
FAW_LSU_S					1146	12.6 (8.0–17.7)
FAW_DUAL_R					1139	16.9 (13.6–20.4)

* Number of insects tested to generate EC_50_. EC_50_ values (ng/cm^2^) were derived from 5-day exposure of BenFAW, BrFAW and FAW_Vip3A_R neonates and 7-day exposure of FAW_LSU_S and FAW_DUAL_R neonates. At the concentration of 3000 ng/cm^2^, 100% of BrFAW neonates (total 72) and 99.2% of FAW_Vip3A_R neonates (total 120) survived and advanced to 2nd instar 5 days after exposure to corresponding Cry1Fa and Vip3Aa19 protein.

## Data Availability

Not applicable.

## Supplementary Materials

The following supporting information can be downloaded at: https://www.mdpi.com/article/10.3390/toxins14120852/s1, Figure S1: FAW feeding assays with eCry1Gb.1Ig-expressing corn events. Three independent events (Event 1, Event 2 and Event 3) of corn carrying *eCry1Gb.1Ig* were used for leaf disc feeding assays that lasted for 3 days. BenFAW: susceptible FAW strain; FAW_Vip3A_R: Vip3Aa-resistant strain; and BrFAW: Cry1Fa-resistant strain, Figure S2: FAW feeding assays with eCry1Gb.1Ig-expressing soybean events. Three independent events (Event 1, Event 2 and Event 3) of soybean carrying *eCry1Gb.1Ig* were used for leaf disc feeding assays that lasted for 3 days. BenFAW: susceptible FAW strain; FAW_Vip3A_R: Vip3Aa-resistant strain; and BrFAW: Cry1Fa-resistant strain.
