# Supplementary material for: eCry1Gb.1Ig, A Novel Chimeric Cry Protein with High Efficacy against Multiple Fall Armyworm (Spodoptera frugiperda) Strains Resistant to Different GM Traits
Source: Toxins (Basel). 2022 Dec 3;14(12):852. doi: 10.3390/toxins14120852 (PMC9785401; doi:10.3390/toxins14120852)
Supplement: Supplementary file 1 [file toxins-14-00852-s001.zip › toxins-2035996-supplementary.pdf]

toxins

Supplementary Materials

eCry1Gb.1Ig, A Novel Chimeric Cry Protein with High Efficacy against Multiple Fall Armyworm (*Spodoptera frugiperda*) Strains Resistant to Different GM Traits

Hyunsook Chae, Zhimou Wen, Travis Hootman, Jo Himes, Qianqian Duan, Joel McMath, Jesse Ditillo, Richard Sessler, Jared Conville, Ying Niu, Phillip Matthews, Fabricio Francischini, Fangneng Huang and Matthew Bramlett

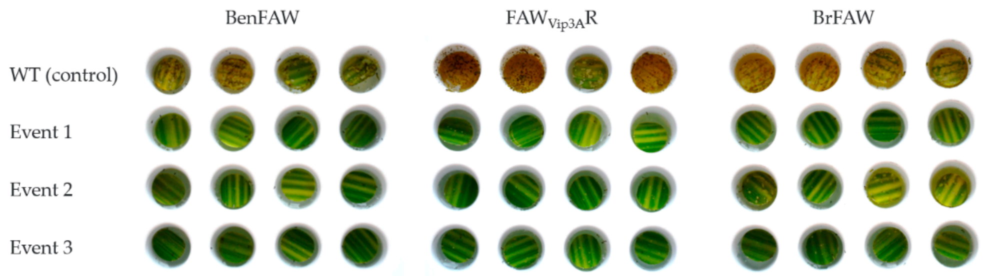

Figure S1. FAW feeding assays with eCry1Gb.1Ig-expressing corn events. Three independent events (Event 1, Event 2 and Event 3) of corn carrying *eCry1Gb.1Ig* were used for leaf disc feeding assays that lasted for 3 days. BenFAW: susceptible FAW strain; $FAW_{Vip3A}R$: Vip3Aa-resistant strain; and BrFAW: Cry1Fa-resistant strain.

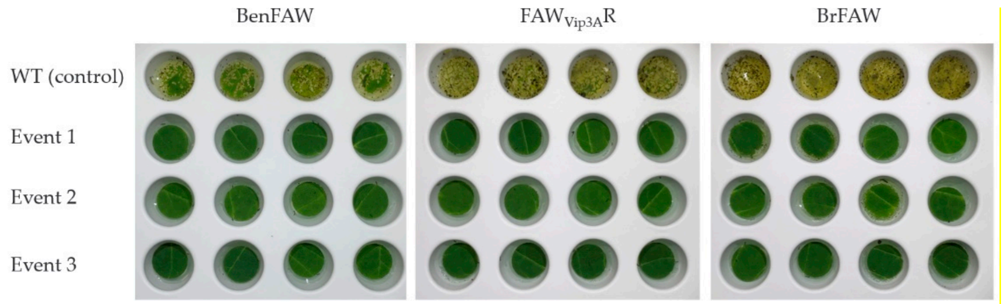

Figure S2. FAW feeding assays with eCry1Gb.1Ig-expressing soybean events. Three independent events (Event 1, Event 2 and Event 3) of soybean carrying *eCry1Gb.1Ig* were used for leaf disc feeding assays that lasted for 3 days. BenFAW: susceptible FAW strain; $FAW_{Vip3A}R$: Vip3Aa-resistant strain; and BrFAW: Cry1Fa-resistant strain.
